# Catechin hydrate suppresses MCF-7 proliferation through *TP53*/*Caspase*-mediated apoptosis

**DOI:** 10.1186/1756-9966-29-167

**Published:** 2010-12-17

**Authors:** Ali A Alshatwi

**Affiliations:** 1 Molecular Cancer Biology Research Lab (MCBRL), Dept. of Food Science and Nutrition, College of Agriculture and Food Sciences, King Saud University, Saudi Arabia

## Abstract

Catechin hydrate (CH), a strong antioxidant that scavenges radicals, is a phenolic compound that is extracted from plants and is present in natural food and drinks, such as green tea and red wine. CH possesses anticancer potential. The mechanism of action of many anticancer drugs is based on their ability to induce apoptosis. In this study, I sought to characterize the downstream apoptotic genes targeted by CH in MCF-7 human breast cancer cells. CH effectively kills MCF-7 cells through induction of apoptosis. Apoptosis was confirmed by terminal deoxynucleotidyl transferase-mediated dUTP nick end labeling (TUNEL) and real-time PCR assays. Cells were exposed to 150 μg/ml CH and 300 μg/mL CH for 24 hours, which resulted in 40.7% and 41.16% apoptotic cells, respectively. Moreover, a 48-hour exposure to 150 μg/ml CH and 300 μg/ml CH resulted in 43.73% and 52.95% apoptotic cells, respectively. Interestingly, after 72 hours of exposure to both concentrations of CH, almost 100% of cells lost their integrity. These results were further confirmed by the increased expression of caspase-3,-8, and -9 and *TP53 *in a time-dependent and dose-dependent manner, as determined by real-time quantitative PCR. In summary, the induction of apoptosis by CH is affected by its ability to increase the expression of pro-apoptotic genes such as caspase-3, -8, and -9 and *TP53*.

## Introduction

Catechin compounds including (-)- epigallocatechin-3-gallate (EGCG), (-)- epigallocatechin (EGC), epicatechin-3-gallate (ECG) and (p)catechin [1] have been shown to exhibit cytostatic properties in many tumor models [2,3]. In addition, the growth of new blood vessels required for tumor growth has been prevented by green tea [4]. In Asian countries, a number of epidemiological observations have suggested that the low incidence of some cancers is due to the consumption of green tea [2,3]. Moreover, epidemiological observations have suggested that the consumption of green tea inhibits growth of many tumor types [5,6].

Breast cancer is the most common cancer and is the leading cause of death for women worldwide [7]. Several epidemiological observations have suggested that increased consumption of green tea is related to improved prognosis of human breast cancer [2] and that the low risk of breast cancer is associated with the intake of green tea in Asian-Americans [8,9]. The modulation of signal transduction pathways, inhibition of cell proliferation, induction of apoptosis, inhibition of tumor invasion and inhibition of angiogenesis are mechanisms that have been established as inhibiting carcinogenesis [10,11]. These potentially beneficial effects of green tea are attributed to catechin compounds, particularly EGCG, which is the most abundant and extensively studied catechin compound of green tea [12,13].

**Figure 1:**
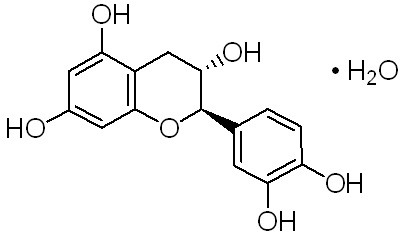
**Molecular structure of catechin hydrate**.

The overall medicinal effects of green tea observed thus far, are focused on combined activities of several compounds in green tea rather than that of a single compound. In addition, most studies have investigated the different synergistic bioactivities of all compounds present in tea extracts or have been focused mainly on the role of EGCG. Therefore, the present study was designed to elucidate the role of the anticancer activity of single compound i.e. CH (Figure 1) at the molecular level.

## Materials and methods

### Catechin Hydrate-A compound of Catechins

Catechin is a polyphenolic flavonoid which has been isolated from a variety of natural sources including tea leaves, grape seeds, and the wood and bark of trees such as acacia and mahogany. Catechin is a more potent antioxidant than ascorbate or α-tocopherol in certain *in vitro *assays of lipid peroxidation. Catechin inhibits the free radical-induced oxidation of isolated LDL by AAPH [14]. Catechins and other related procyanidin compounds have antitumor activity when tested in a two-stage mouse epidermal carcinoma model employing topical application. Following is the structure of (+)-Catechin hydrate.

### Preparations of CH

100 mg CH was dissolved in 10 mL DMEM medium (10% FCS) to obtain stock solution and was further diluted in medium to obtain desired concentrations.

### Maintenance of MCF-7 Cells

The MCF-7 breast cancer cell line was a kind gift from Dr. M. A. Akbarshah at the Mahatma Gandhi-Doerenkamp Center (MGDC) for Alternatives to Use of Animals in Life Science Education, Bharathidasan University, India. The cell line was maintained and propagated in 90% Dulbecco's Modified Eagle's Medium (DMEM) containing 10% fetal bovine serum (FBS) and 1% penicillin/streptomycin. Cells were cultured as adherent monolayers (i.e., cultured at ~70% to 80% confluence) and maintained at 37°C in a humidified atmosphere of 5% CO2. Cells were harvested after being subjected to brief trypsinization. All chemicals used were of research grade.

### Viability of Cells

Cell viability was assayed using a trypan blue exclusion test as explained earlier with slight modifications[15].

### Toxicity and Cell Proliferation Assays

The Cell Titer Blue^® ^viability assay (Promega Madison, WI) was performed to assess the toxicity of different concentrations of CH on MCF-7 cells. The assay was performed according to the manufacturer's instructions. Briefly, MCF-7 cells (2 × 10^4 ^cells/well) were plated in 96-well plates and treated with 0 μg/mL CH and 160 μg/mL CH for 24 hours. Then, 40 μL of the Cell Titer Blue solution was directly added to the wells and incubated at 37°C for 6 hours. The fluorescence was recorded with a 560 nm/590 nm (excitation/emission) filter set using a Bio-Tek microplate fluorescence reader (FLx800™), and the IC_50 _was calculated. Quadruplet samples were run for each concentration of CH in three independent experiments.

### CH Treatment for a concentration- and Time-Dependent Study

For a concentration- and time-dependent study, two sets of CH concentrations (50 μg/mL and 150 μg/mL; 300 μg/mL and 600 μg/mL) were considered for treatment of MCF-7 cells for 24 hours. I found that 50 μg/mL CH did not show any significant induction of apoptosis whereas 600 μg/mL CH completely killed the cells. Hence, 150 μg/mL and 300 μg/mL concentrations of CH were used for further studies.

MCF-7 cells were treated with either 150 μg/mL or 300 μg/mL CH for 24, 48 and 72 hours for the terminal deoxynucleotidyl transferase-mediated dUTP nick end labeling (TUNEL) assay. The cells were incubated with the same CHconcentrations for 24 and 48 hours for real-time quantitative PCR analysis.

### TUNEL Assay

The DeadEnd^® ^TUNEL assay kit (Promega, Madison, WI) was used for studying apoptosis in a time- and dose-dependent manner. The manufacturer's instructions were followed with slight modifications. Briefly, MCF-7 cells (1.5 × 10^6 ^cells/well) were cultured in 6-well plates to study apoptosis in adherent cells. Cells were treated with 150 μg/mL and 300 μg/mL CH for 24, 48 and 72 hours. After the incubation period, the culture medium was aspirated off, and the cell layers were trypsinized. The trypsinized cells were reattached on 0.01% polylysine-coated slides, fixed with 4% methanol-free formaldehyde solution, and stained according to the DeadEnd fluorometric TUNEL system protocol [16]. The stained cells were observed using a Carl-Zeiss (Axiovert) epifluorescence microscope using a triple band-pass filter. To determine the percentage of cells demonstrating apoptosis, 1000 cells were counted in each experiment [17].

### Real-time quantitative PCR analysis

The expression of apoptotic genes was analyzed by reverse transcription-PCR (RT-PCR; Applied Biosystems 7500 Fast, Foster City, CA) using a real-time SYBR Green/ROX gene expression assay kit (QIAgen). The cDNA was directly prepared from cultured cells using a Fastlane^® ^Cell cDNA kit (QIAGEN, Germany), and the mRNA levels of *Caspase 3*, *Caspase 8*, *Caspase 9 *and *tp53 *as well as the reference gene, *GAPDH*, were assayed using gene-specific SYBR Green-based QuantiTect^® ^Primer assays (QIAGEN, Germany). Quantitative real-time RT-PCR was performed in a reaction volume of 25 μL according to the manufacturer's instructions. Briefly, 12.5 μL of master mix, 2.5 μL of primer assay (10×) and 10 μL of template cDNA (100 μg) were added to each well. After a brief centrifugation, the PCR plate was subjected to 35 cycles of the following conditions: (i) PCR activation at 95°C for 5 minutes, (ii) denaturation at 95°C for 5 seconds and (iii) annealing/extension at 60°C for 10 seconds. All samples and controls were run in triplicates on an ABI 7500 Fast Real-time PCR system. The quantitative RT-PCR data was analyzed by a comparative threshold (Ct) method, and the fold inductions of samples were compared with the untreated samples. GAPDH was used as an internal reference gene to normalize the expression of the apoptotic genes. The Ct cycle was used to determine the expression level in control cells and MCF-7 cells treated with CH for 24 and 48 h. The gene expression level was then calculated as described earlier [18]. The results were expressed as the ratio of reference gene to target gene by using the following formula: ΔCt = Ct (apoptotic genes) - Ct (GAPDH). To determine the relative expression levels, the following formula was used: ΔΔCt = ΔCt (Treated) - ΔCt (Control). Thus, the expression levels were expressed as n-fold differences relative to the calibrator. The value was used to plot the expression of apoptotic genes using the expression of 2^-ΔΔCt^.

## Results

### Effect of CH on MCF-7 breast cancer cell proliferation and apoptosis

To explore the anticancer effect of CH on MCF-7 human breast cancer cells, several in vitro experiments were conducted.

### Viability assay

The viability of cells was greater than 95%.

### Determination of CH toxicity on MCF-7 cells

**Figure 2:**
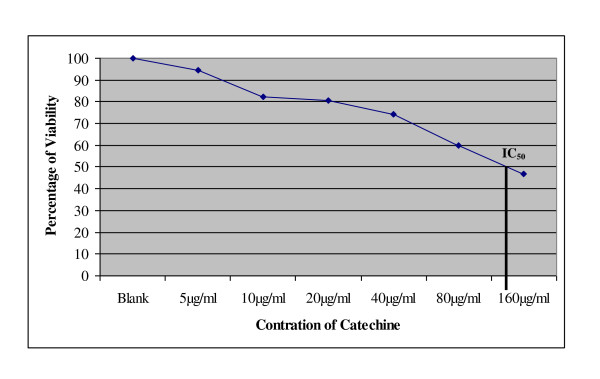
**Determination of IC_50 _of catechin against the MCF-7 breast cancer cell line**.

The cytotoxic effect of 0 μg/mL CH and 160 μg/mL CH on MCF-7 cells was examined using the Cell Titer Blue^® ^viability assay (Promega Madison, WI). A dose-dependent reduction in color was observed after 24 hours of treatment with CH, and 54.76% of the cells were dead at the highest concentration of CH tested (160 μg/mL) whereas the IC_50 _of CH was achieved at 127.62 μg/mL CH (Figure 2).

### Quantification of apoptosis by a TUNEL assay

**Figure 3:**
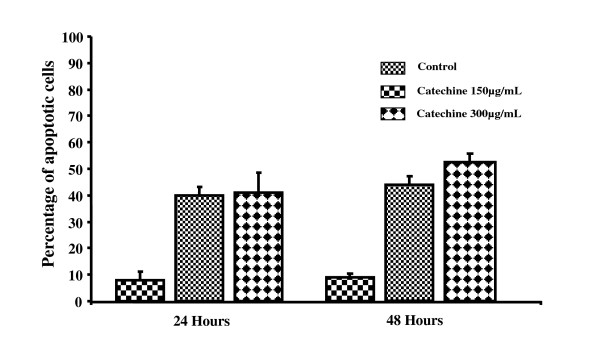
**Percentage of apoptotic cells in 24 hours and 48 hours incubation in blank control and treatments with catechin hydrate (150 μg/mL and 300 μg/mL)**.

**Figure 4:**
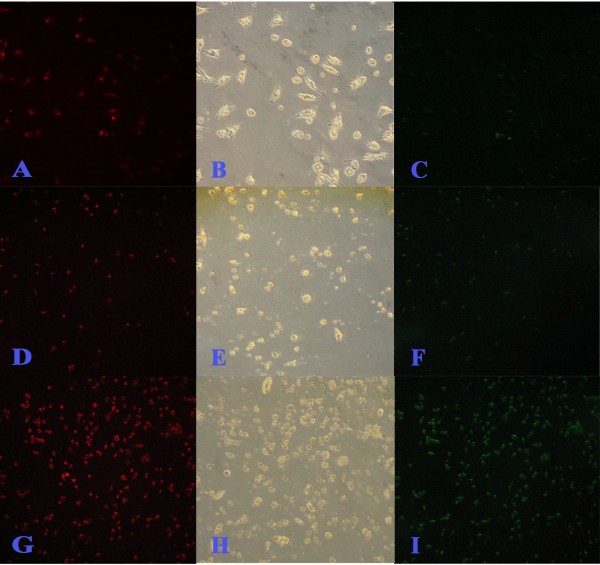
**TUNEL assay (microscopic) after 24 hours incubation of MCF-7 against catechine treatment**. A, B and C are untreated control; D, E and F treated with 150 μg/mL of catechine; G, H and I treated with 300 μg/mL of catechine. Red fluorescence is due to Propedium Iodide staining and observed under green filter while green fluorescence is due to FITC staining and observed under blue filter. Bright field image (B, E and H) central row. Observations done at 200× magnification.

**Figure 5:**
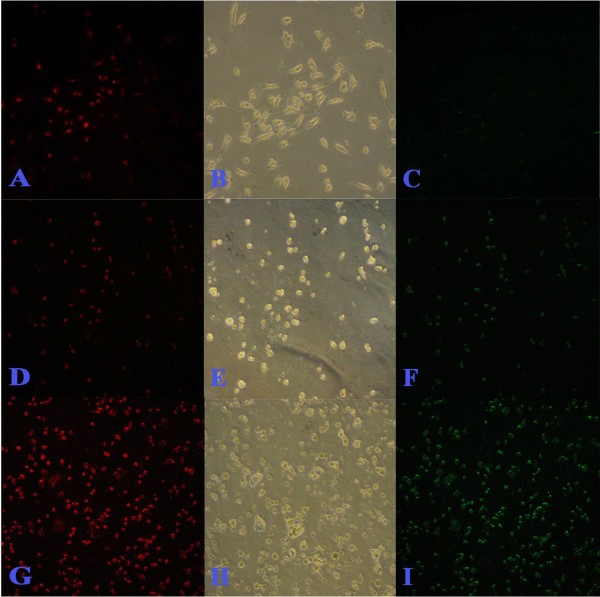
**TUNEL assay (microscopic) after 48 hours incubation of MCF-7 against catechine treatment**. A, B and C are untreated control; D, E and F treated with 150 μg/mL of catechine; G, H and I treated with 300 μg/mL of catechine. Red fluorescence is due to Propedium Iodide staining and observed under green filter while green fluorescence is due to FITC staining and observed under blue filter. Bright field image (B, E and H) central row. Observations done at 200× magnification.

**Figure 6:**
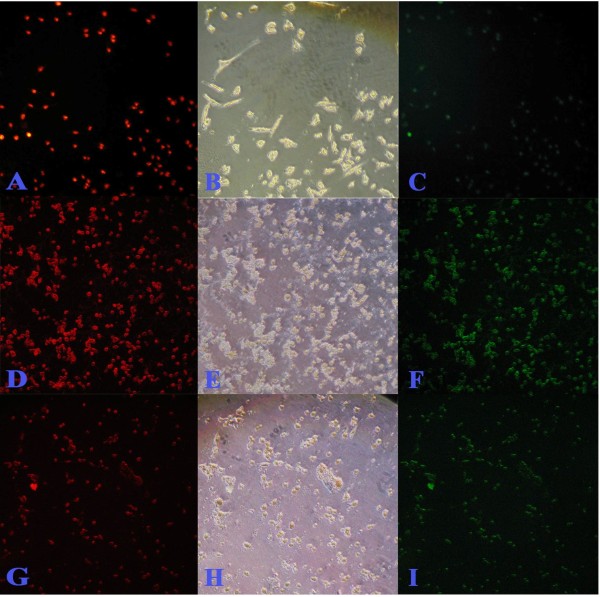
**TUNEL assay (microscopic) after 72 hours incubation of MCF-7 against catechine treatment**. A, B and C are untreated control; D, E and F treated with 150 μg/mL of catechine; G, H and I treated with 300 μg/mL of catechine. Red fluorescence is due to Propedium Iodide staining and observed under green filter while green fluorescence is due to FITC staining and observed under blue filter. Bright field image (B, E and H) central row. Observations done at 200× magnification.

To determine whether the inhibition of cell proliferation by CH was due to the induction of apoptosis, a TUNEL assay was used. Figures 3, 4, 5 and 6 summarize the effect of CH on MCF-7 cells. A dose- and time-dependent increase in the induction of apoptosis was observed when MCF-7 cells were treated with CH. When compared to the control cells at 24 hours, 40.7 and 41.16% of the cells treated with 150 μg/mL and 300 μg/mL CH, respectively, underwent apoptosis. Similarly, 43.73 and 52.95% of the cells treated with 150 μg/mL and 300 μg/mL CH, respectively, for 48 hours underwent apoptosis. Interestingly, after 72 hours of exposure to CH, almost 100% of the cells in both concentrations had lost their integrity (Figure 6).

### Quantification of mRNA levels of apoptotic-related genes

To investigate the molecular mechanism of CH-induced apoptosis in MCF-7 cells, the expression levels of several apoptosis-related genes were examined. The relative quantification of *Caspase-3*, *-8*, and *-9 *and *Tp53 *mRNA expression levels was performed by SYBR Green-based quantitative real-time PCR (RT-PCR) using a 7500 Fast Real Time System (Applied Biosystems).

**Figure 7:**
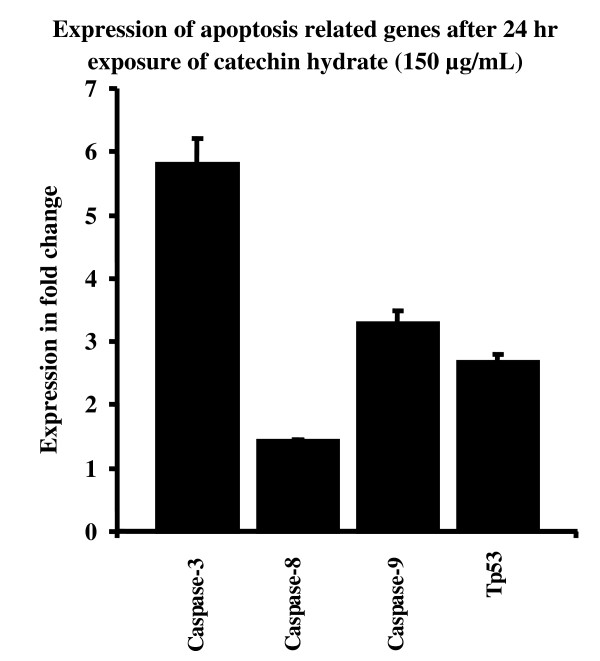
**Comparision of chang in expression of apoptosis related genes as fold change (ratio of target:reference gene) in MCF-7 cells after 24 hours of exposure of 150 μg/mL of catechin**.

**Figure 8:**
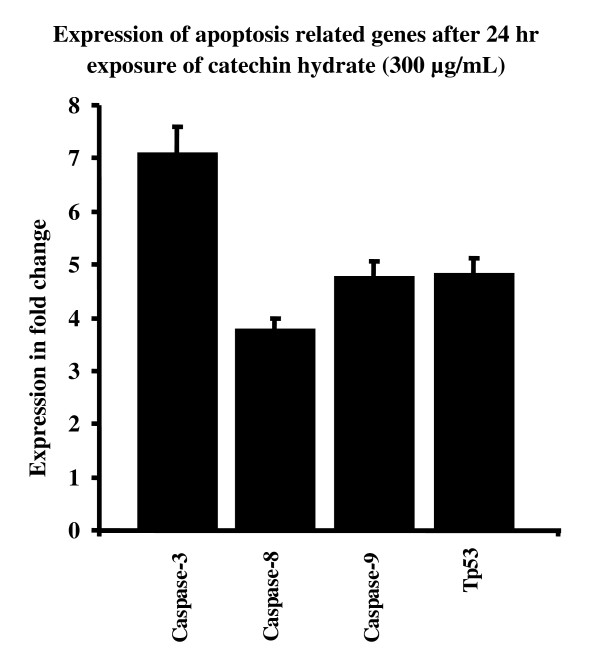
**Comparision of chang in expression of apoptosis related genes as fold change (ratio of target:reference gene) in MCF-7 cells after 24 hours of exposure of 300 μg/mL of catechin**.

**Figure 9:**
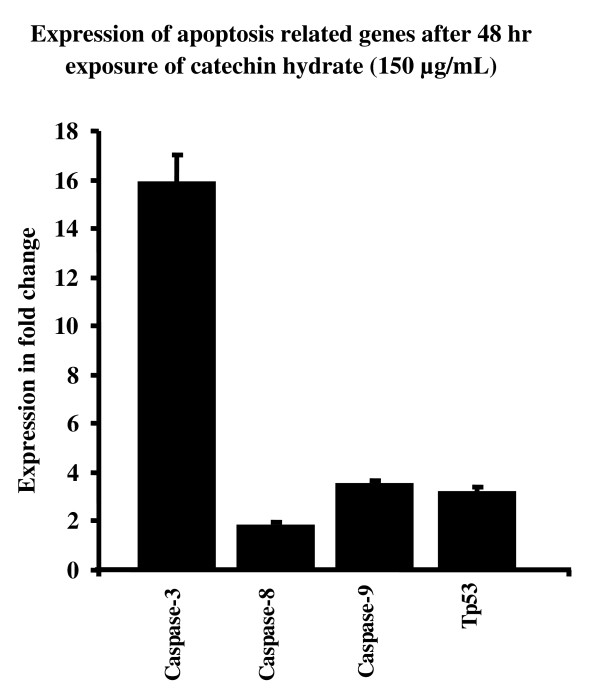
**Comparision of chang in expression of apoptosis related genes as fold change (ratio of target:reference gene) in MCF-7 cells after 48 hours of exposure of 150 μg/mL of catechin**.

**Figure 10:**
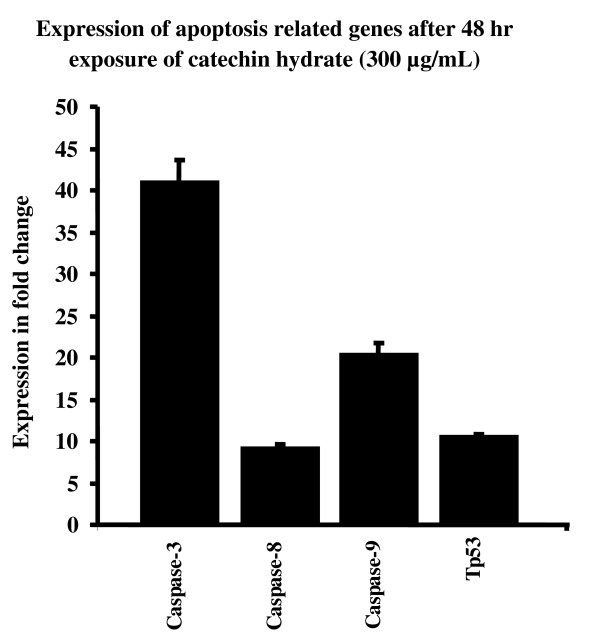
**Comparision of chang in expression of apoptosis related genes as fold change (ratio of target:reference gene) in MCF-7 cells after 48 hours of exposure of 300 μg/mL of catechin**.

Figures 7 to 10 summarize the gene expression changes of *Caspase-3*, *-8*, and *-9 *and *p53*. CH increased the transcripts of *Caspase 3*, *-8*, and *-9*, and *p53 *by several fold. The expression levels of these genes in MCF-7 cells treated with 150 μg/ml CH for 24 h increased by 5.81, 1.42, 3.29, and 2.68 fold, respectively, as compared to the levels in untreated control cells (Figure 7). Similarly, the expression levels of *Caspase-3*, -*8*, and -*9 *and *p53 *in MCF-7 cells treated with 300 μg/ml CH for 24 h increased by 7.09, 3.8, 478, and 4.82 fold, respectively, as compared to levels in untreated control cells (Figure 8). In a time-dependent manner, the expression levels of the apoptotis-related genes in MCF-7 cells treated with 150 or 300 μg/ml CH for 48 h increased when compared to the levels in untreated control cells (Figure 9 and 10). However, the expression levels of *Caspase-3*, *-8*, and *-9 *and *p53 *in MCF-7 cells treated with 300 μg/ml CH for 48 h markedly increased--40.52, 8.72, 20.26 and 10 fold--as compared to control untreated cells (Figure 10). Together, these data suggest that these caspases and p53 were induced by CH in a dose- and time-dependent manner.

## Discussion

The mechanism of action of many anticancer drugs is based on their ability to induce apoptosis [19,20]. There are many mechanisms through which apoptosis can be enhanced in cells. Agents suppressing the proliferation of malignant cells by enhancing apoptosis may constitute a useful mechanistic approach to both cancer chemoprevention and chemotherapy. However, unfavorable side effects and resistance of many of the anticancer agents that have been developed are serious problems [21]. Thus, there is a growing interest in the use of plant-based compounds to develop safe and more effective therapeutic agents for cancer treatment [22]. Because the side effects of green tea are modest and well tolerated [23], increasing attention is being given to the application of tea catechins for cancer prevention and treatment. EGCG conjugated with capric acid has been shown to be the catechin that most potently induces apoptosis in U937 cells. C10 has been shown to enhance apoptosis in human colon cancer (HCT116) cells [24]. Catechin compounds have been shown to exhibit cytostatic properties in many tumor models [2,3]. Babich et al. (2005) found that catechin and epicatechin (EC) are less toxic than other catechin compounds, including ECG, CG, EGCG and EGC, in HSC-2 carcinoma cells and HGF-2 fibroblasts[25]. Hence, I was interested in identifying whether apoptosis was the mode of death for cancer cells treated with CH (the least toxic form). To do so, I sought to determine the role of CH in inhibiting cell growth and modulating the expression of caspases-3, -8, and -9 and p53.

The data presented in this paper demonstrate a time- and dose-dependent inhibition by CH of MCF-7 human breast cancer cell proliferation. There are many mechanisms through which apoptosis can be induced in cells. The sensitivity of cells to any of these stimuli may vary depending on factors such as the expression of pro- and anti-apoptotic proteins. The mitochondrial apoptotic pathways and death receptor pathways are the two major pathways that have been characterized in mammalian cells. The mitochondria have a central role in regulating the caspase cascade and apoptosis [26]. Caspases have a central role in the apoptotic process in that they trigger a cascade of apoptotic pathways [27]. The release of cytochrome -c from mitochondria leads to the activation of procaspase-9 and then caspase-3 [26]. The activation of caspase-3 is an important downstream step in the apoptotic pathway [28]. In addition, the effector caspase, caspase-3, and the initiator caspases, caspase-8 and -9, are the main executors of apoptosis [29]. Caspase-8 is in the death receptor pathway whereas caspase-9 is in the mitochondrial pathway, and both pathways share caspase-3 [30]. Treatment with EGCG conjugated with capric acid increases the formation of reactive oxygen species (ROS), loss of mitochondrial membrane potential (MMP), release of cytochrome c, activation of caspase-9 and activation of caspase-3. In addition, EGCG conjugated with capric acid also activates the extrinsic pathway as demonstrated by the time-dependent increase in Fas expression and caspase-8 activity [24]. Two distinct downstream pathways have been identified for activation of apoptosis after caspase-8 is activated. In one pathway, caspase-8 directly processes downstream effector caspase-3, -6, and -7. In an alternative pathway, caspase-8 activates crosstalk between the death receptor pathway and the mitochondrial pathway by the cleavage of Bid to Bid, a pro-apoptotic member of the Bcl2 family. The activation of caspase-8 has a central role in Fas-mediated apoptosis. Moreover, the cleavage of Bid has been shown to be associated with caspase-8 activation [31]. Taken together, the data presented in this study suggest that catechin-induced apoptosis is mediated by the death receptor and mitochondrial apoptotic pathways as demonstrated by increased expression levels of caspase-3, -8 and -9 after CH treatment. In addition, this study suggests that catechin activates the extrinsic death pathway as demonstrated by increased expression levels of caspase-8.

p53, the most commonly mutated gene associated with cancer [32], helps to regulate the cell cycle and has a key role in ensuring that damaged cells are destroyed by apoptosis. The data presented in this study indicate that the expression levels of p53 and caspase-3, -8 and -9 were markedly increased after CH treatment in a concentration-dependent manner. These data suggest that catechin induced apoptosis by regulating pro-apoptotic genes.

The possibility that p53-mediated apoptosis may be associated with the activation of caspase-3, -8 and -9 is suggested by the ability of p53 to activate both the extrinsic and intrinsic apoptotic pathways [30,33,34]. p53 enhances cancer cell apoptosis, and it prevents cell replication by stopping the cell cycle at G1 or interphase [35]. By inducing the release of mitochondrial cytochrome c, p53 might be able to activate effector caspases including caspase-3. Caspase-3, -8, and -9 may be the apoptotic effector machinery engaged by p53 to mediate teratogen-induced apoptotic pathways [36].

## Conclusion

In conclusion, to our knowledge, the results presented in this study show for the first time that CH exhibits anticancer effects by blocking the proliferation of MCF7 cells and inducing apoptosis in part by modulating expression levels of caspase-3, -8, and -9 and p53. The induction of apoptosis by CH is affected by its ability to regulate the expression of pro-apoptotic genes such as caspase-3, -8, and -9 and p53. Taken together, it is most likely that CH induced, at least in part, p53 and caspase-mediated apoptosis in MCF-7 cells. Therefore, the present study demonstrates that CH significantly inhibits the growth of MCF-7 human breast cancer cells in vitro, and it provides the underlying mechanism for the anticancer activity. CH suppressed the growth of breast cancer cells without significant toxicity, making it a promising chemotherapeutic agent for breast cancer treatment; this is likely to be confirmed by further investigation.

## Competing interests

The author declares that they have no competing interests.
